# Growing our eye care service: the story of Visualiza Eye Care System

**Published:** 2022-12-16

**Authors:** Mariano Yee Melgar

**Affiliations:** 1 Founder: Visualiza Eye Care System, Guatemala.

**Appropriate technology and an attitude of service were key factors in Visualiza’s improved cataract output.**

**Figure:**
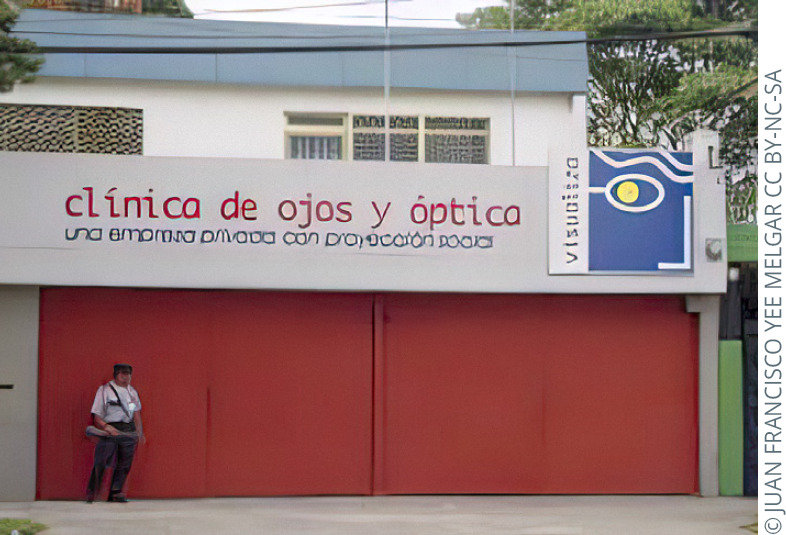
Visualiza in 1998. **GUATEMALA**

**Figure:**
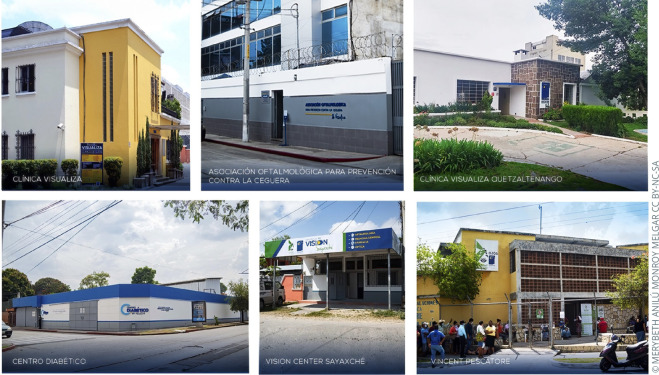
Visualiza in 2022. **GUATEMALA**

What should you do when a blind person comes to your clinic and has no means to pay for your services? Could you leave this person in the dark?

These are some of the questions we faced in our early years as a small, private eye clinic, with only two ophthalmologists and two employees, carrying out just 67 cataract operations a year in 1998. We wanted to grow, and help more people, but we had many questions. For example, how could we help people, and still be sustainable? How could we reach the most vulnerable groups of people in our area?

In 2002, we decided to start an eye care model that had never existed in Latin America before: a private practice that offered subsidised care to those unable to pay for services. Now, two decades later, we have 227 employees, including 24 ophthalmologists and 20 optometrists, working in two main hospitals and eight vision centres, performing over 4,000 cataract operations and 6,000 other procedures a year.

## How we got here

In the beginning, we did a lot of planning and set goals for ourselves, the first of which was to become self-supporting (financially sustainable) within the first six months. We also communicated with our staff members regularly to ensure everyone shared the same values. When you want to serve patients, it’s important to create a culture where the people working for you feel connected; where they think about what it means to come to work. Then they will come to the clinic to change people’s lives; to serve and give their best.

We were not afraid to evaluate ourselves and to change our ways of working, if needed. We recognised that we could only improve if we introduced protocols and standardised all our procedures (not only the clinical and surgical procedures), and if we kept records of everything we did. It is easier to detect problems in the system when you know exactly what everyone is doing! Because we kept accurate records, we could measure how many new patients were coming in, how many were coming for follow-up visits, the acceptance rate for our services, how many outreach activities we provided (and how effective they were), the financial status of the service, and so on. This allowed us to track our progress and continue to improve.

For example, in the beginning, the proportion of patients who accepted surgery to restore or preserve their vision was just 30%. In order to increase the acceptance rate, we introduced a counsellor. This was a local person, trained by us, who explained everything that the patient needed to know about a surgical procedure, with patience and care, and in a way that was easy for the patient to understand. By using good communication skills, the counsellor helps to reinforce the doctor’s recommendations and increases compliance. As a result, 65% of patients who are offered surgery now agree to go ahead with it. The counsellor is one of our most valuable team members!

## Appropriate technology

We use appropriate technology. While everybody around us was doing phacoemulsification, we started using the manual small-incision cataract surgical technique we learnt at Aravind Eye Care System in India. The results were amazing: postoperative visual acuity was as good as with phacoemulsification, but at less than 25% of the cost. Manual small-incision cataract surgery is affordable because it doesn’t require special machines and equipment. It also helps to increase the number of patients you can serve, because you can carry out the procedure in any standard operating room at a minimal cost for the patient. We have so much confidence in this method that it was my method of choice when I performed cataract surgery on my own mother.

**Figure:**
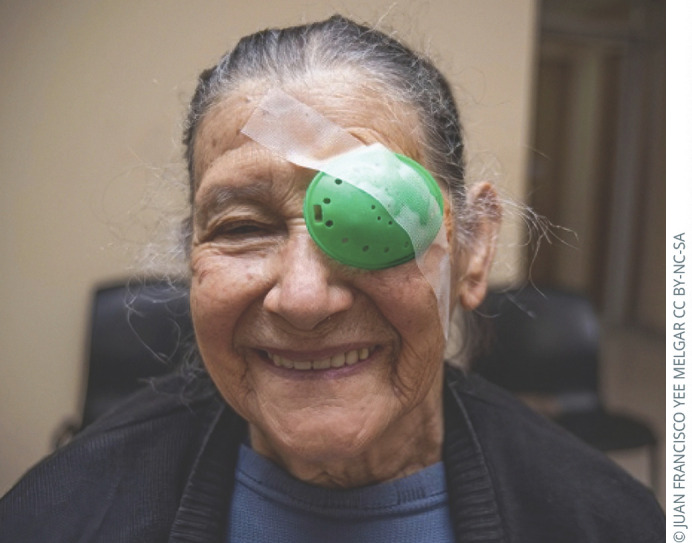
A patient after cataract surgery at Visualiza Eye Care System. **GUATEMALA**

**Figure:**
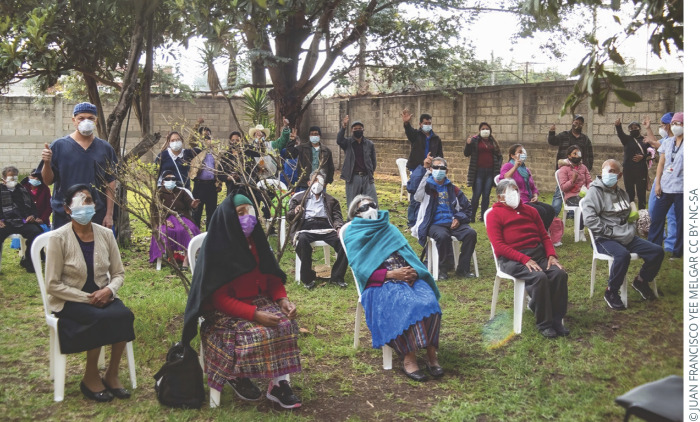
Cataract patients received surgery during an outreach expedition in the Quetzaltenango area. **GUATEMALA**

Because the cost per operation was lower, and we could carry out more operations; this created additional income that helped us to grow and diversify by introducing other services, such as refractive error and diabetes services. Diabetes is becoming a big problem in Guatemala, so we established a deparment for diabetes patients where there are nutritionists, endocrinologists, and retinal specialists. This has generated even more income, which means we can now provide outreach services in areas where eye care is not easily accessible.

## Outreach

Outreach is a key component of our strategy. It is a great marketing technique and an effective tool for reaching those most in need. We visit different places to find patients who don’t have access to eye care and might need cataract surgery or spectacles, or who have other eye conditions. Recently, we trained our staff members in sign language and we are now receiving hearing-impaired people as well. Having happy patients makes everything easier.

In the future, we would like to establish even more eye hospitals and vision centres across the nation so we can continue to provide high quality eye care to all.

**Keys to success**

- A focus on patients, not profits will bring in more patients, thereby increasing the number of operations you can perform (your output). This will lead to greater financial sustainability.
- Higher output levels mean higher quality surgery (practice makes perfect). Good outcomes makes for happier patients who will recommend your services to friends and family members.
- Build a team that believes in what you are doing.
- Choose surgical methods that are affordable for patient; appropriate technology is affordable technology.
- Outreach is the key to reaching patients who would otherwise never have surgery.
- Constantly evaluate yourself and your organisation. Ask yourself: Where are we now? What can we do better? Is my service good enough for me and for my own family?

